# Assessing the Feasibility of Using a Multiplex Serological Assay to Conduct Serosurveillance for Malaria Exposure in Deployed Military Personnel

**DOI:** 10.3390/tropicalmed10010013

**Published:** 2025-01-02

**Authors:** Sidhartha Chaudhury, Jessica S. Bolton, Edwin Kamau, Elke S. Bergmann-Leitner

**Affiliations:** 1 Bacterial and Parasitic Diseases Department, Walter Reed Army Institute of Research-Armed Forces Research Institute of Medical Sciences, Bangkok 10400, Thailand; sidhartha.chaudhury.mil@afrims.org; 2 Biologics Research & Development Branch, Walter Reed Army Institute of Research, Silver Spring, MD 20910, USA; jessica.s.bolton.ctr@health.mil; 3 Tripler Army Medical Center, Honolulu, HI 96859, USA; edwin.kamau.mil@health.mil

**Keywords:** *Plasmodium*, exposure, mosquito saliva, serological profile, electro-chemiluminescence, biomarker, multiplex assay

## Abstract

Reproducibly assessing malaria exposure is critical for force health protection for military service members deployed to malaria-endemic regions as well as for civilians making public health decisions and evaluating malaria eradication efforts. However, malaria disease surveillance is challenged by under-reporting, natural immunity, and chemoprophylaxis, which can mask malaria exposure and lead to an underestimation of malaria prevalence. In this study, we determined the feasibility of using a serosurveillance-based approach to measure Anopheles vector exposure, Plasmodium sporozoite exposure, and blood-stage parasitemia using a multiplex serological panel. We tested post-deployment samples obtained from U.S. service members returning from regions with malaria risk to assess the potential of this serosurveillance panel. The results identified that some service members had anti-CSP antibody levels comparable to those found in endemic populations, suggesting exposure to sporozoites while those individuals were on chemoprophylaxis. We also observed isolated cases of anti-MSP1 levels that were as high as those observed in endemic populations and in CHMI studies, suggesting possible cases of clinical or subclinical parasitemia. The study demonstrated the feasibility of implementing a multiplex serology approach for conducting serosurveillance for Anopheles vector exposure and Plasmodium parasite exposure in samples collected following military deployments and its potential to support public health policies.

## 1. Introduction

Malaria remains a persistent threat to military forces deploying to malaria-endemic regions worldwide. While chemoprophylaxis is widely prescribed, prophylaxis failure is still observed due to issues with poor or sporadic compliance, drug resistance (reviewed in [1]), and limitations of standard prophylaxis regimens in preventing infection from hypnozoite-forming *Plasmodia* such as *P. vivax* [2]. Given the significant operational impact of malaria in military deployments to endemic regions, having timely information on malaria risk and exposure during and after deployments is critical for military medical planners to make appropriate decisions regarding force health protection.

Monitoring the prevalence of disease vectors involves the use of a range of conventional, globally recommended entomological surveillance tools. Unfortunately, these tools have notable shortcomings: they are labor intensive and costly; rely on traps with variable efficacy; are highly variable due to abiotic factors (e.g., humidity, wind, temperature, and rainfall); and do not always correlate with exposure to mosquito bites or disease, especially in the presence of interventions such as insecticide-treated clothing or bed nets. Sampling methods like human landing catches raise concerns related to oversight requirements, ethical issues, and operator biases.

The traditional means by which malaria risk is assessed is through passive disease surveillance, whereby clinically documented malaria cases, either within a deployed military unit or among the local population are used to determine malaria risk. However, such surveillance is confounded by under-reporting and natural immunity among local populations and the use of vector exposure prevention methods and/or chemoprophylaxis by military personnel, both of which serve to mask the true extent of malaria exposure and lead to an underestimate of malaria risk. In this study, we sought to assess the feasibility of using a multiplex serology assay that measures antibody responses to the *Anopheles* vector and *Plasmodium* antigens to measure vector and malaria exposure in deployed service members.

Multiplex serological testing platforms have increased sensitivity and specificity by defining serological profiles of test samples [3,4,5] based on multiple antigens simultaneously. Prior studies have shown that measuring antibody responses to mosquito saliva proteins, such as gSG6 (e.g., *An. gambiae* gSG6-P1), in residents living in endemic areas reliably correlates with vector exposure and can be used to evaluate entomological interventions including mass deployment of insecticidal bed nets [6,7] and ivermectin, mass-drug administration [8]. Likewise, measuring antibody responses to *Plasmodium* sporozoite-antigens, such as CSP, and blood-stage antigens, such as AMA1 and MSP-1, has previously been used to detect exposure to sporozoites and blood-stage parasites, respectively [9]. In a previous study using a multiplex panel containing both *Anopheles* and *Plasmodium* antigens, we observed a direct relationship between exposure to vectors and the pathogen at both an individual and community level, indicating the malaria serosurveillance can supplement or even replace conventional passive-surveillance methods [9]. Serosurveillance in military operations differs in several ways from public health serosurveillance including that military personnel are often serologically naïve to endemic pathogens; they are smaller in number than local populations, and they are highly mobile.

The present study explores the feasibility of conducting serosurveillance of antibodies directed against the mosquito saliva protein gSG6 and an array of *Plasmodial* antigens in deployed U.S. military service members. Exposure to mosquito saliva (via insect bite) will result in the induction of mosquito saliva-specific Ab [9] with a direct correlation between efficacy of countermeasure and level of mosquito saliva-specific antibodies. The simultaneous presence of antibodies to the sporozoite stage antigens of *Plasmodium* and the absence of antibodies to blood-stage antigens can indicate efficacy of chemoprophylaxis and anti-malarial drugs.

## 2. Materials and Methods

### 2.1. Samples

The use of clinical samples has been reviewed by WRAIR human subjects protection branch (HSPB) and approved (exempt human use protocols (WRAIR#xx). Samples analyzed in this study were obtained from various sources (Table 1): Sera from military service members deployed to one of eight African countries were from the Department of Defense Serum Repository (DoD SR, approved usage WRAIR#2593) [10]. The study sought to assess the feasibility of running the assay on a large number of samples collected from military deployment and thus there were no other specific selection criteria. Malaria-naïve sera were from healthy U.S. donors with no history of traveling to geographic regions with malaria through a blood collection protocol (WRAIR#2567) and a commercial source (Gemini Bio Products, West Sacramento, CA, USA) and served as negative controls. Sera from non-vaccinated, malaria-naïve participants of a controlled human malaria infection (CHMI [11]) and sera from individuals vaccinated with irradiated sporozoites delivered by mosquito bite (IMRAS, WRAIR#2036) (www.clinicaltrials.gov NCT01994525) were used as positive controls to validate the assay [12]. Malaria-endemic samples were obtained from study-participants enrolled in a prospective study of acute HIV-1 infections in East Africa (Kenya and Uganda) [9,13,14].

We analyzed samples with diverse malaria exposure histories to assess the feasibility of using a multiplex serological assay to conduct serosurveillance and detect recent malaria exposure (Table 1). For baseline measurements, we used 30 samples from U.S. malaria-naïve donors. To assess serological responses following known exposure to *Plasmodium falciparum*, we used samples from individuals immunized with irradiated sporozoites (IMRAS) via mosquito bite, and individuals enrolled in CHMI studies conducted at the Walter Reed Army Institute of Research (WRAIR). IMRAS samples represent a *Plasmodium* infection with irradiated sporozoites that does not progress beyond the liver stage of infection. CHMI participants were naïve individuals exposed to five infectious bites by mosquitoes infected with *P. falciparum* that was allowed to progress to blood-stage infection before treatment with anti-malarial drugs. We also analyzed samples collected from individuals living in the malaria-holoendemic countries of Kenya (Kericho, moderate endemicity) and Uganda (Kampala, high endemicity). Since the original sample collection was not focused on malaria, we argue that the sample distribution was unbiased, and therefore representing the broader population in that given geographic region. Finally, we applied that assay to 755 samples from the U.S. Dept of Defense serum repository (DoD SR). These samples were collected from U.S. military personnel deployed to the sub-Saharan African countries of Chad, Djibouti, Ethiopia, Kenya, Liberia, Mali, Niger, Somalia, Kenya, and Uganda. All samples were collected within three months of returning from deployment.

### 2.2. Antigens

Recombinant *P. falciparum* (3D7 strain) proteins (MSP-1, AMA-1, Pfs25, Pfs16, CelTOS, GTP-binding protein (GBP), ETRAMP4, and ETRAMP5) were produced at Genscript (Piscataway, NJ, USA). Peptides derived from the *Anopheles gambiae* salivary gland protein (gSG6; peptides 1 and 2 [15]), the *Plasmodium falciparum* circumsporozoite protein (CSP; representing the major repeat NANP [16], and the C-terminus [17]) were synthesized by Atlantic Peptides (Lewisburg, PA, USA).

We applied the in-house developed multiplex antigen panel assessing the presence of the antibodies to Anopheles vector salivary antigens, pre-erythrocytic and blood stage antigens for *P. falciparum*, and bovine serum albumin (BSA) as a negative control (summarized in Table 2). The gSG6 *Anopheles* salivary antigens can provide an indication of the level of vector exposure [9,18]. Antibody responses to *P.f.* pre-erythrocytic sporozoite antigens provide an indication of the level of sporozoite exposure, potentially even in the presence of chemoprophylaxis that would prevent blood-stage infection. Antibodies to *P.f.* blood-stage antigens may serve as serological markers for the level of blood-stage parasite exposure. The persistence of antibodies to the respective *Plasmodial* antigens differs significantly, thereby allowing conclusions on when the last exposure had occurred. For example, antibodies to the repeat region of the CSP, ETRAMP4, and ETRAMP5 have been shown to be relatively short-lived thus serving as a marker of recent infections [19]. Similarly, antibodies to mosquito saliva proteins, such as gSG6, are short-lived, waning over the course of 4–6 weeks [20] and, therefore, present an opportunity to assess the interval between serum collection and last exposure to the vectors.

### 2.3. Electro-Chemiluminescence Immunoassay (ECLIA) 

Assay development included rationale for the plate antigens, and defining the sensitivity of the respective plate antigens in identifying seroconversion has been previously reported [9]. The agreement with conventional serological assays, such as the ELISA, has also been assessed [21,22]. The multiplex ECLIA methodology deployed for this study is based on the MesoScale U-PLEX platform utilizing 10-spot ECLIA plates (MSD, Gaithersburg, MD, USA) and performed as described previously [21]. Briefly, biotinylated proteins were diluted to a concentration of 300 nM using coating diluent (1× PBS with 0.5% BSA) and linked with a unique U-plex linker provided by the U-PLEX platform (MSD), vortexed, and incubated at room temperature (RT) for 30 min. The U-PLEX-coupled protein solutions were brought up to 6 mL with Stop Solution, creating a 1× multiplex coating solution. Plates were coated with the cocktail of proteins and incubated at RT for 1 h on a Titramax plate shaker (Heidolph, Schwabach, Germany), shaking at 700 rpm. Coated plates can be stored for up to seven days at 2–8 °C based on the manufacturer’s protocol. After incubation, the plates were washed with a working solution of 1× MSD Wash Buffer (MSD) three times. Sera were diluted to the desired concentration with Diluent 2 (MSD) added to each well and incubated at RT for 1 h on a plate shaker. Plates were washed three times with 1× MSD Wash Buffer and incubated with detection antibody, SULFO-TAG goat anti-human antibody (diluted to 1 µg/mL in Diluent 3 (MSD).Plates were sealed and incubated at RT for 1 h on a plate shaker (700 rpm). After washing, MSD Read Buffer T was added to each well and the plates were read on the MESO QuickPlex SQ 120 reader (MSD), per the manufacturer’s instructions.

### 2.4. Statistical Analysis

The MSD assay provides a readout in units of mean luminescence intensity, and all readouts were directly log-transformed prior to analysis. Univariate analysis comparisons between groups were made using a Shapiro–Wilk Normality Test followed by a Student’s T-test or a Wilcoxon signed rank test. We applied a multiple test correction using the Benjamini–Hochberg method; *p*-values were considered significant if their adjusted *p*-value was <0.01. All statistical analysis was carried out in R using the stats, *ggplot2*, and *corrplot* packages.

## 3. Results

### 3.1. Markers of Malaria Exposure

**Figure 1:**
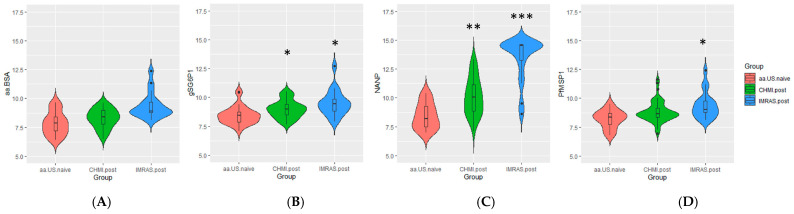
Antibody responses to panel antigens in U.S. malaria-naïve individuals (red), CHMI (green), and IMRAS (blue) subjects for BSA (**A**), the *Anopheles* salivary antigen gSG6P1 (**B**), the *Sporozoite* antigen CSP (**C**), and the blood-stage antigen MSP1 (**D**). Statistically significant differences are shown as *p* < 0.01 (*), *p* < 0.001 (**), and *p* < 1 × 10^−4^ (***).

We applied the multiplex serology assay to U.S. malaria-naïve samples, IMRAS samples, and CHMI samples to identify which antigens could serve as a sensitive marker for malaria exposure. Responses to two antigens, the central repeat region of the circumsporozoite surface protein (CSP(NANP)) and the merozoite surface protein-1 (MSP1) showed significant differences between the IMRAS and CHMI, and the malaria-naïve controls (Figure 1). There were no statistically significant differences in the reactivity to BSA indicating that batch effects or group level difference in background or non-specific binding were minimal. For gSG6p1, CHMI subjects and IMRAS subjects showed modest but significantly (*p* < 0.01) higher antigen responses than naïve controls. For CSP(NANP), the CHMI group showed significantly higher responses than the US naïve group (*p* < 1 × 10^−3^), with an average of five-fold higher titers; the IMRAS group also showed significantly higher responses compared to the U.S. malaria-naïve group (*p* < 1 × 10^−4^) with an average of 160-fold higher titers. For MSP1, CHMI subjects showed on average two-fold higher titers than the US malaria-naïve group, but the difference was not statistically significant. IMRAS subjects showed statistically significant three-fold higher titers than the US malaria-naïve group (*p* < 0.01). No other antigens in the panel showed significant differences between CHMI or IMRAS and the US malaria naïve group.

### 3.2. Defining Thresholds for Seropositivity

We sought to define the threshold for seropositivity that considers inter- and intra-group variability. First, we assessed the inter- and intra-group variability in the U.S. naïve, CHMI, and IMRAS groups as well as the in the DoD SR sample set for each antigen in the panel in terms of both the average standard deviation within the groups (σ_intra_), and the standard deviation of the means of each group (σ_group_). Supplementary Table S1 lists the variability results for each antigen in the panel. Overall, we found that the differences in means between the groups was minimal, with an average difference of 0.23 in log scale, which is equivalent to a 1.25-fold titer range. Mean intergroup variability was 0.48, corresponding to an approximately two-fold titer range. Mean intra-group variability was 1.1, equivalent to an approximately three-fold titer range. To determine a threshold for seropositivity, we defined a standard deviation that accounts for both inter- and intra-group variability as (σ_combined_). We then defined the threshold corresponding to >99.9% of a normal distribution centered on the mean value from the U.S. malaria-naïve samples, with a standard deviation of σ_combined_, which corresponds to a z-score of 2.87 for each antigen:

$$\sigma_{combined}=\sqrt{\sigma_{group}^{2}+\sigma_{intra}^{2}}\;threshold=\mu_{naive}+2.87*\sigma_{combined}$$

The resulting cutoff values are shown in Supplementary Table S1. Overall, 20- to 70-fold higher titers over the average titers in the U.S. naïve samples are needed for the response to be classified as seropositive with a theoretical 99.9% specificity for this antigen panel.

### 3.3. Assay Sensitivity and Specificity

We measured the sensitivity and specificity of the assay for detecting malaria exposure in CHMI and IMRAS subjects compared to U.S. malaria-naïve subjects, where all CHMI and IMRAS were considered “malaria exposed” and all U.S. malaria-naïve subjects were considered “not exposed”. For this analysis, we varied the cutoff threshold for seropositivity from 8 to 15 and assessed sensitivity and specificity. Overall, at lower cutoffs, sensitivity would be expected to be high as more exposed subjects are classified as seropositive (true positives), but specificity would be expected to be low, as more non-exposed subjects are also classified as seropositive (false negatives). Conversely, at high cutoff values, the sensitivity would be low, as more exposed subjects are classified as seronegative (false negatives) and the specificity would be high, as more non-exposed subjects are classified as seronegative (true negatives). We analyzed sensitivity and specificity for the responses to CSP(NANP) and MSP1 antigens because they were the only two antigens to show substantially higher antibody responses (greater than two-fold) in CHMI and IMRAS samples compared to U.S. naïve samples.

For CSP(NANP) responses, using a threshold cutoff value of 12.6 (Supplementary Table S1) corresponding to a theoretical 99.9% specificity, we observed 65% sensitivity for malaria exposure in IMRAS subjects and a 10% sensitivity for malaria exposure in CHMI subjects. For MSP1, using a threshold cutoff value of 11.32 (Supplementary Table S1), we observed 0% sensitivity of malaria exposure in IMRAS and 4% sensitivity for malaria exposure in CHMI. The sensitivity plot (Supplementary Figure S1) for CSP (NANP) shows that moderate to high sensitivity (60 to 80%) for IMRAS-level malaria exposure is possible at a wide range of cutoffs, but sensitivity for CHMI malaria exposure is moderate to low (<30%). For MSP1, sensitivity is low for both CHMI and IMRAS (<10%).

### 3.4. Seropositivity for Malaria Exposure

We next sought to determine the level of malaria exposure in the Kenyan and Ugandan endemic samples collected from the RV217 study and the DoD SR samples, both in terms of seropositivity for CSP(NANP) and MSP-1 (cutoff-dependent approach) and in terms of any observed group-level differences in mean antibody responses (cutoff-independent approach). First, we evaluated CSP (NANP) from CHMI and IMRAS studies where subjects had a 21% and 83% seropositivity, respectively, both which were significantly higher mean titers than the U.S. malaria-naïve subjects (*p* < 1 × 10^−4^ and *p* < 0.001, respectively). Next, we analyzed clinical study samples from Kenya (Kericho—region of moderate endemicity) and Uganda (Kampala - high endemicity), in which we found seropositivity rates of 7% and 23%, respectively. The Uganda group average responses were significantly higher than the U.S. malaria-naïve responses and comparable to CHMI and IMRAS samples (*p* < 1 × 10^−4^). Finally, we applied the assay to the DoD SR samples and found relative low rates of seropositivity, with 5% in Chad, 3% in Djibouti, 2% in Liberia, 2% in Niger, and 2% in Somalia.

**Figure 2:**
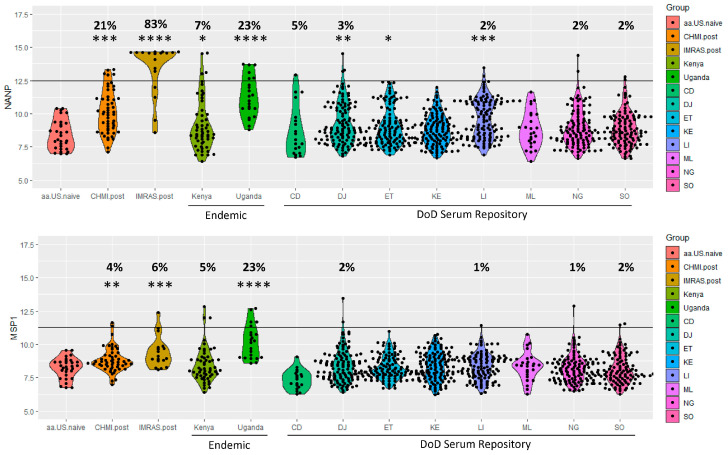
Seropositivity for CSP(NANP) and MSP-1 antigens in samples from endemic countries and the DoD Serum Repository (SR). Antibody responses are shown for U.S. malaria-naïve samples, CHMI and IMRAS samples, samples from malaria endemic populations in Kenya and Uganda, and DoD SR samples. Seropositivity % is reported as well as the statistical significance of any group-level differences in responses with that of U.S. naïve samples. Country abbreviations: CD = Chad, DJ = Djibouti, ET = Ethiopia, KE = Kenya, LI = Liberia, ML = Mali, NG = Niger, SO = Somalia. Statistical significance: *p* < 0.05 (*), *p* < 0.01 (**), *p* < 0.001 (***), and *p* < 1 × 10^−4^ (****).

CSP(NANP) responses are indicative of sporozoite exposure, which can occur even in the presence of chemoprophylaxis. We previously observed that MSP-1 responses, overall, have relatively low sensitivity, but given the high specificity, we sought to determine the seropositivity rate in this data set (Figure 2). For CHMI and IMRAS subjects, we observed a seropositivity of 4 and 6%, respectively, but we did see that the group mean average for these two groups was significantly higher than for U.S. malaria-naïve samples (*p* < 0.001 and *p* < 0.01, respectively). For Kenyan endemic samples, we similarly saw a seropositivity rate of 5%. For Ugandan endemic samples, we saw a seropositivity of 23% and a significantly higher group response compared to U.S. malaria-naïve samples (*p* < 1 × 10^−4^). This likely reflects the high endemicity of malaria in Uganda, and the high likelihood of adults in the region having a history of repeated prior malaria episodes. Among the DoD SR samples, we observed several isolated cases of seropositivity in samples of service members returning from Djibouti, Liberia, Niger, and Somalia, suggesting that some of them were exposed while deployed despite routine military preventive measures.

### 3.5. Necessary Sample Size for Serosurveillance

Given the moderate sensitivity of the assay for detecting malaria exposure based on antibody responses to CSP(NANP) and MSP-1 antigens, we sought to determine the sample size needed to implement this assay for serosurveillance purposes. We used the CHMI and IMRAS sample set as surrogates for malaria infection as the true sensitivity of this assay as detection of natural malaria exposure is not known. As such, we carried out a power analysis to determine the sample size needed to reject the null hypothesis (incidence rate < 1%) with an alpha of 0.05 and a power of 0.80 at a range of true incidence rates at three different theoretical assay sensitivities: 5% (corresponding to the relatively low level of exposure we see in CHMI cases), 70% (the relatively high level of exposure we see in IMRAS cases), and 20% (an intermediate level of sensitivity). In a real-world scenario with high malaria endemicity (incidence rate of >20%) assuming high assay sensitivity (70% such as in IMRAS), a sample size of 60 would be sufficient to reliably measure malaria exposure; assuming a low assay sensitivity (5% such as in CHMI), a sample size of 800 would be required, while assuming a moderate sensitivity (20%, in-between CHMI and IMRAS), a sample size of 110 would be required.

## 4. Discussion

The present study investigates the performance of a multiplex-antigen serological assay panel in detecting exposure to *Anopheles* mosquitoes and *Plasmodium* parasites. Our results show that this serological panel can reliably detect 20–50-fold increases in antibodies titers from baseline responses (i.e., U.S. malaria-naïve) and was successfully applied to 755 samples from the DoD SR from U.S. service members previously deployed to African countries. Analyzing the antibody levels to the sporozoite-antigen CSP(NANP) revealed evidence of exposure to *Plasmodium* sporozoites while presumably under chemoprophylaxis, as this is standard policy under the U.S. Africa Combatant Command (USAFRICOM). The multiplex panel demonstrated its utility of (1) measuring exposure to *Plasmodium* to determine malaria risk even under chemoprophylaxis; (2) measuring the effectiveness of drugs to prevent blood-stage malaria; and potentially (3) determining the exposure rate to vectors associated with malaria transmission.

We found that returning U.S. service members from African countries such as Djibouti and Liberia had significantly higher anti-CSP(NANP) titers compared to U.S. naïve individuals consistent with exposure to *Plasmodium* infected mosquitoes and successful use of chemoprophylactic drugs. Some of the service members tested in this study had anti-CSP(NANP) titers comparable to levels found in endemic populations indicating high transmission intensity of malaria. Similarly, only a few service members had MSP-1 antibody levels as high as endemic populations, suggesting possible cases of clinical or subclinical parasitemia (breakthrough infections). While we do not have access to the medical history of the samples, it is possible that these individuals were diagnosed with malaria.

More studies are needed on serological response in natural infection, particularly in a naïve population, to determine the sensitivity of the panel for use in serosurveillance and the added value of the other *Plasmodium* antigens in informing on recent exposure to sporozoites vs. subclinical infections. Here, the sensitivity analysis was based on two different non-natural forms of *Plasmodium* infection: CHMI and IMRAS. From the serological data, CHMI resulted in relatively low levels of serological response to NANP and MSP-1. For CSP(NANP), a single five-bite exposure of sporozoites (likely delivering fewer than 1000 sporozoites [23,24]) may not be sufficient to induce strong anti-CSP antibody responses, compared to repeated exposure over time in a natural infection setting. For MSP-1 response, low responses may observed because blood-stage parasitemia in CHMI is cut short by close monitoring, frequent testing, and immediate treatment upon detection, limiting the blood-stage parasite burden and likely the corresponding serological response. For IMRAS, subjects are given a massive cumulative dose of 810–1235 bites (up to 80,000 sporozoites) [12], many orders of magnitude more than the <1000 sporozoites in CHMI. This large dose of sporozoites may be responsible for the extremely high anti-CSP responses seen in this group, even compared to the high-endemicity Uganda samples. We would expect natural exposure and infection is likely to fall somewhere in between these two models, more immunogenic than CHMI (for blood stage antigens) but not as immunogenic as IMRAS (for pre-erythrocytic antigens).

Antibodies to mosquito saliva have been shown to be short-lived [15], thereby functioning as a reliable marker of recent mosquito exposure. However, except for the CHMI samples, none of the samples in this study were likely collected within four weeks of mosquito exposure, limiting the applicability of this sample set for entomological surveillance. Future plans include testing samples regularly collected during prospective studies and trials to further define the sensitivity of the multiplex panel.

Malaria continues to impact military and peacekeeping operations in endemic regions, particularly in sub-Saharan Africa as demonstrated by a retrospective analysis of a cumulative total of 295 cases of doxycycline failure in French soldiers returning from the Ivory Coast from 1993 to 2012 [25]. More recently, mefloquine is the chemoprophylaxis of choice for UN peace-keeping operations in Africa. However, mefloquine prophylaxis failure was reported by Guatemalan soldiers deployed to the Democratic Republic of Congo in 2012 [26] and Peruvian soldiers deployed to the Central African Republic in 2016 [27]. Most recently, in 2023, a Royal Thai Army unit of 273 soldiers experienced 64 cases of malaria after a 12-month deployment to South Sudan (manuscript in preparation). The malaria parasites in these cases were *P. falciparum*, *P. vivax*, and *P. ovale*. The factors underlying these cases of prophylaxis failure are unclear and may include inadequate drug compliance or limitations of existing chemoprophylaxis regimens in preventing relapsing malarias, but the issue underscores the importance of ongoing malaria surveillance during these missions.

Beyond military applications, multiplex serology assays have also been explored for use in public health malaria control and elimination programs. Our assay could be used in a serosurveillance program to monitor villages or towns in at-risk regions to assess exposure to *Plasmodium* antigens and guide public health interventions such as vector control and/or targeted or mass drug administration. However, more research is needed to evaluate the feasibility of this assay in the context of malaria control and elimination.

## 5. Conclusions

A serological method for assessing vector and parasite exposure in these missions will be critical to informing future guidelines on malaria prevention and treatment. While the sensitivity of the current panel may not yet be sufficient for detecting low level endemic exposure, it could be used to detect potentially high levels of exposure in deployed settings in highly endemic areas, where failures in vector control measures or chemoprophylaxis compliance could have a significant operational impact. Our findings suggest that this assay could theoretically be used to assess malaria exposure in small units (company-size or larger) deployed to areas of high endemicity, where potentially over 20% or more of the unit could be exposed to malaria during deployment, but further study is needed to demonstrate its feasibility in real-world military settings. Current research efforts are assessing the potential of the assay to inform on efficacy of spatial and topical repellants and thus demonstrate its usefulness in standard testing for public health programs (e.g., National Malaria Control Programs).

## Figures and Tables

**Table 1:** Overview of sample types for serosurveillance testing.

GROUP	Description	Source	Samples (n)
US Naive	US Naïve	WRAIR#2567, commercial	30
IMRAS	Irradiated sporozoite vaccine	NCT01994525 [12]	18
CHMI	Controlled malaria challenge	WRAIR#2593	52
Chad	U.S. military, Post-deployment	DoD serum repository	19
Djibouti	U.S. military, Post-deployment		119
Ethiopia	U.S. military, Post-deployment		121
Kenya	U.S. military, Post-deployment		125
Liberia	U.S. military, Post-deployment		119
Mali	U.S. military, Post-deployment		25
Niger	U.S. military, Post-deployment		116
Somalia	U.S. military, Post-deployment		111
Kenya	Endemic samples	RV217 [14]	55
Uganda	Endemic samples		22

**Table 2:** Antigens included in multiplex testing panel for serosurveillance.

Antigen	Species	Description
BSA	negative control	negative control
gSG6P1	*Anopheles* spp.	An. gambiae salivary protein
gSG6P2	*Anopheles* spp.	An. gambiae salivary protein
PfTRAP	*P. falciparum*	Sporozoite-stage
NANP	*P. falciparum*	CSP, sporozoite-stage (central repeat)
CSP_Pf16	*P. falciparum*	CSP, sporozoite-stage (C-terminus)
PfCelTOS	*P. falciparum*	sporozoite-stage
AMA	*P. falciparum*	Asexual blood-stage
PfMSP1	*P. falciparum*	Asexual blood-stage
Pfs16	*P. falciparum*	Sexual blood-stage
Pfs25	*P. falciparum*	Sexual blood-stage
ETRAMP4	*P. falciparum*	Asexual blood-stage
ETRAMP5	*P. falciparum*	Asexual blood-stage
GBP	*P. falciparum*	Asexual blood-stage
Rifin	*P. falciparum*	Asexual blood-stage

## Data Availability

All data are contained within the manuscript. R scripts can be obtained from the contributing author upon request.

## Supplementary Materials

The following supporting information can be downloaded at: https://www.mdpi.com/article/10.3390/tropicalmed10010013/s1, Figure S1: Sensitivity and specificity in detection malaria exposure in CHMI, IMRAS, and U.S. naïve subjects using antibody response to CSP(NANP) and MSP-1 antigens; Figure S2: Sample size calculation for measuring a range of malaria exposure rates based on an assay sensitivity of 5%, 20%, and 70%. Table S1: Inter- and intra-group variability and seropositivity cutoff values.
