# Supplementary material for: Assessing the Feasibility of Using a Multiplex Serological Assay to Conduct Serosurveillance for Malaria Exposure in Deployed Military Personnel
Source: Trop Med Infect Dis. 2025 Jan 2;10(1):13. doi: 10.3390/tropicalmed10010013 (PMC11768601; doi:10.3390/tropicalmed10010013)
Supplement: Supplementary file 1 [file tropicalmed-10-00013-s001.zip › tropicalmed-3368275-supplementary.pdf]

Table S1. Inter- and intra-group variability and seropositivity cutoff values

Antigen	Baseline Mean	σ_{group}	σ_{intra}	$\sigma_{combined}$	Cutoff Value	Titer Fold Change from baseline for cutoff value
gSG6P1	8.44	0.46	0.97	1.08	11.54	22.10
gSG6P2	7.89	0.47	0.97	1.08	11.00	22.39
PfTRAP	8.29	0.39	1.09	1.15	11.60	27.36
NANP	8.43	0.31	1.42	1.45	12.60	64.58
CSP_Pf16	7.71	0.43	0.93	1.03	10.66	19.02
PfCelTOS	8.02	0.47	0.99	1.09	11.16	22.96
AMA	7.95	0.44	0.89	1.00	10.80	17.41
PfMSP1	8.22	0.50	0.96	1.08	11.32	22.26
Pfs16	7.92	0.35	1.01	1.07	11.00	21.62
Pfs25	7.86	0.46	1.03	1.12	11.08	25.09
ETRAMP4	7.65	0.39	0.95	1.02	10.58	18.78
ETRAMP5	8.49	0.38	1.15	1.21	11.96	32.10
GBP	9.20	0.21	1.03	1.05	12.22	20.59
Rifin	7.83	0.30	1.00	1.05	10.84	20.36

Supplementary data Chaudhury et al.

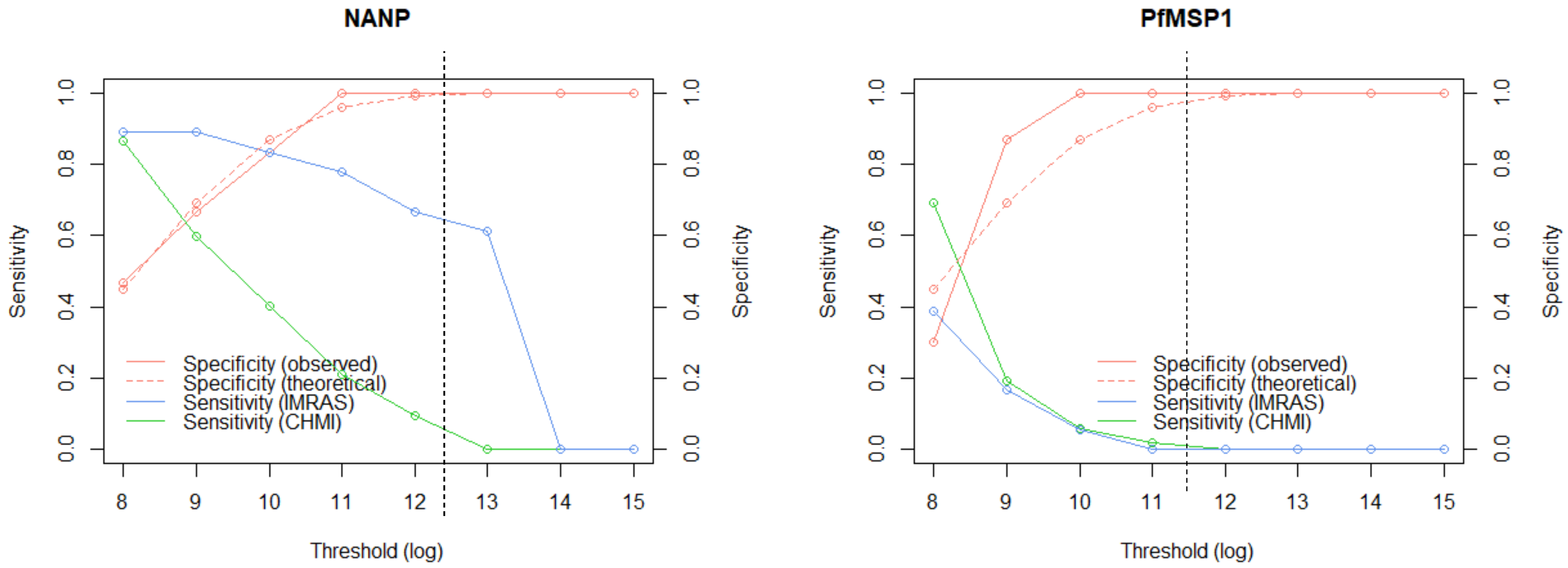

Figure S1. Sensitivity and specificity in detection malaria exposure in CHMI, IMRAS, and U.S. naïve subjects using antibody response to CSP(NANP) and MSP-1 antigens. Specificity is shown as both the observed specificity from the U.S. malaria-naïve data, and the theoretical specificity based on a normal distribution with a standard deviation ($\sigma_{combined}$) and baseline mean for CSP(NANP) and MSP1 from Table S1.

Supplementary data Chaudhury et al.

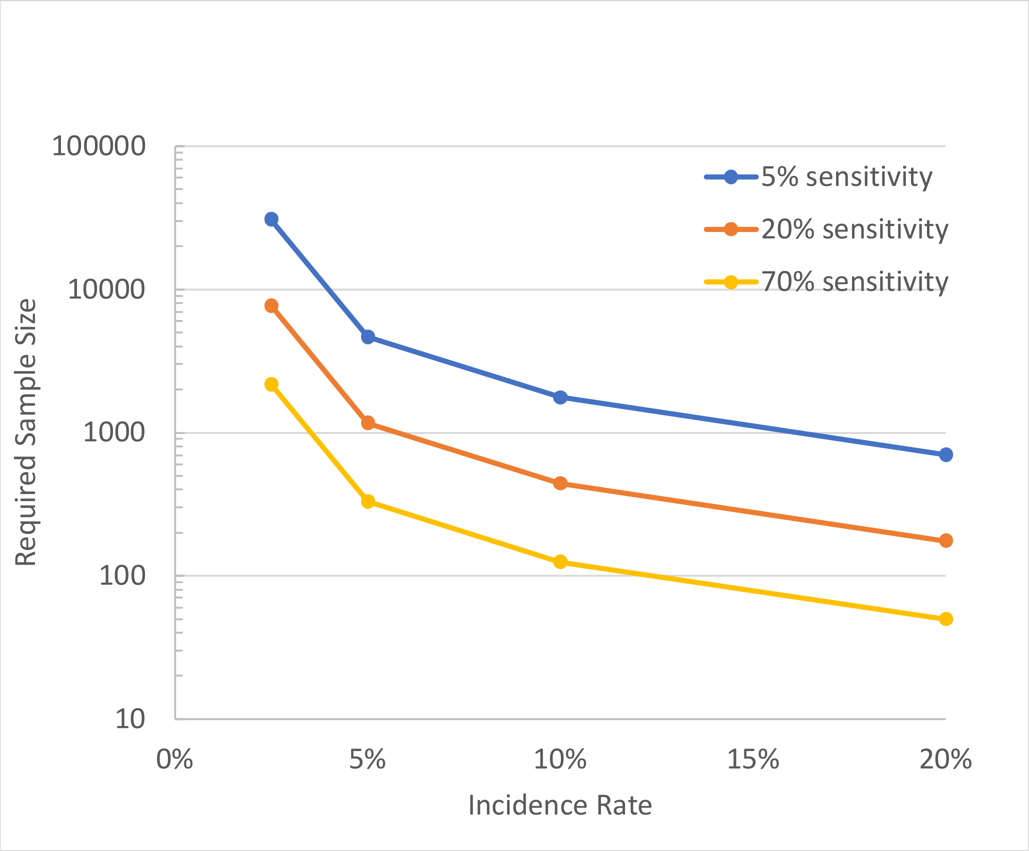

Figure S2. Sample size calculation shows the minimum sample size needed to reliably detect a non-zero incidence rate at a range of malaria incidents rates (from 0% to 20%) based on an assay sensitivity of 5% (blue), 20% (red orange), and 70% (yellow). Power analysis assumes an alpha of 0.05 and a power of 0.80."
